# Nuancing the need for speed: temporal health system strengthening in low-income countries

**DOI:** 10.1136/bmjgh-2019-001816

**Published:** 2019-08-30

**Authors:** Tom Bashford, Alexis Joannides, Kamal Phuyal, Santosh Bhatta, Julie Mytton, Robert Harrison, Peter Hutchinson

**Affiliations:** 1 NIHR Global Health Research Group for Neurotrauma, University of Cambridge, Cambridge, UK; 2 NIHR Global Health Research Group on Burn Trauma, Kathmandu, Nepal; 3 NIHR Global Health Research Group on Nepal Injury Research, University of the West of England Bristol, Bristol, UK; 4 NIHR Global Health Research Group on African Snakebite Research, Liverpool School of Tropical Medicine, Liverpool, UK

**Keywords:** health systems, traumatology, burns, snake bite, stings and other envenoming

**Summary box**

- Delays in receiving care are of particular relevance to time-critical pathologies, for which quality of care and timely access are fundamentally interlinked.
- Characterising and improving delays in a health system are complex, and require both quantitative and qualitative understanding.
- There is mutual benefit to collaboration across clinical, academic and geographical areas of interest in order to understand and reduce delays in accessing care.

Patients with delayed access to medical care often experience worse outcomes. The ‘three delays’ model developed in the context of emergency obstetric care is an important conceptual device for researchers and policy-makers, particularly in resource-poor health systems.^1^ This model characterises delay in terms of (1) the decision to seek care; (2) arrival at a health facility and (3) the provision of adequate care.

However, ‘access’ is a nuanced term, one that is not simply an issue of geographical resource distribution or population density. A patient may seek care, but be constrained by competing demands or health beliefs. Once sought, the care delivered may be inappropriate. Even after arrival at a healthcare facility that is able to deliver the necessary care, there may still be a clinically significant delay in obtaining it.^2^

Furthermore, there may be variable prevailing sociocultural attitudes to different conditions, with a biomedical model of time-critical pathology interacting with multiple other narratives.^3^ Access to care by victims of snakebite may be hampered by a cultural belief that the bite is ‘a manifestation of witchcraft or deity displeasure’.^4^ Alternatively, in the context of neurotrauma, the religious significance of the date for a planned operation may mandate that the procedure be delayed, even after patients and their families are made aware that this could be detrimental to the outcome (unpublished data).

Clearly, quantifying the presence and effect of a delay, while an important step, is only descriptive; improvement mandates a deeper understanding. Delays in accessing care, either in the community or once in an appropriate centre, can arise from a myriad of reasons—financial, logistical, political, procedural and cultural.^5^ For time-critical pathologies, such as neurotrauma, burns, polytrauma and snakebite, systems strengthening requires these issues to be accounted for alongside the clinical services required to deliver definitive treatment. In 2018, a systematic review in *The Lancet* by Kruk *et al* 6 suggested that ‘access is no longer the only binding constraint for improving survival in low-income and middle-income countries—health system quality must be improved simultaneously’. We would go further to suggest that, at least for certain pathologies, considering access as a different entity to quality is a false dichotomy: good quality care is meaningless if access to it is not timely.

**Figure 1:**
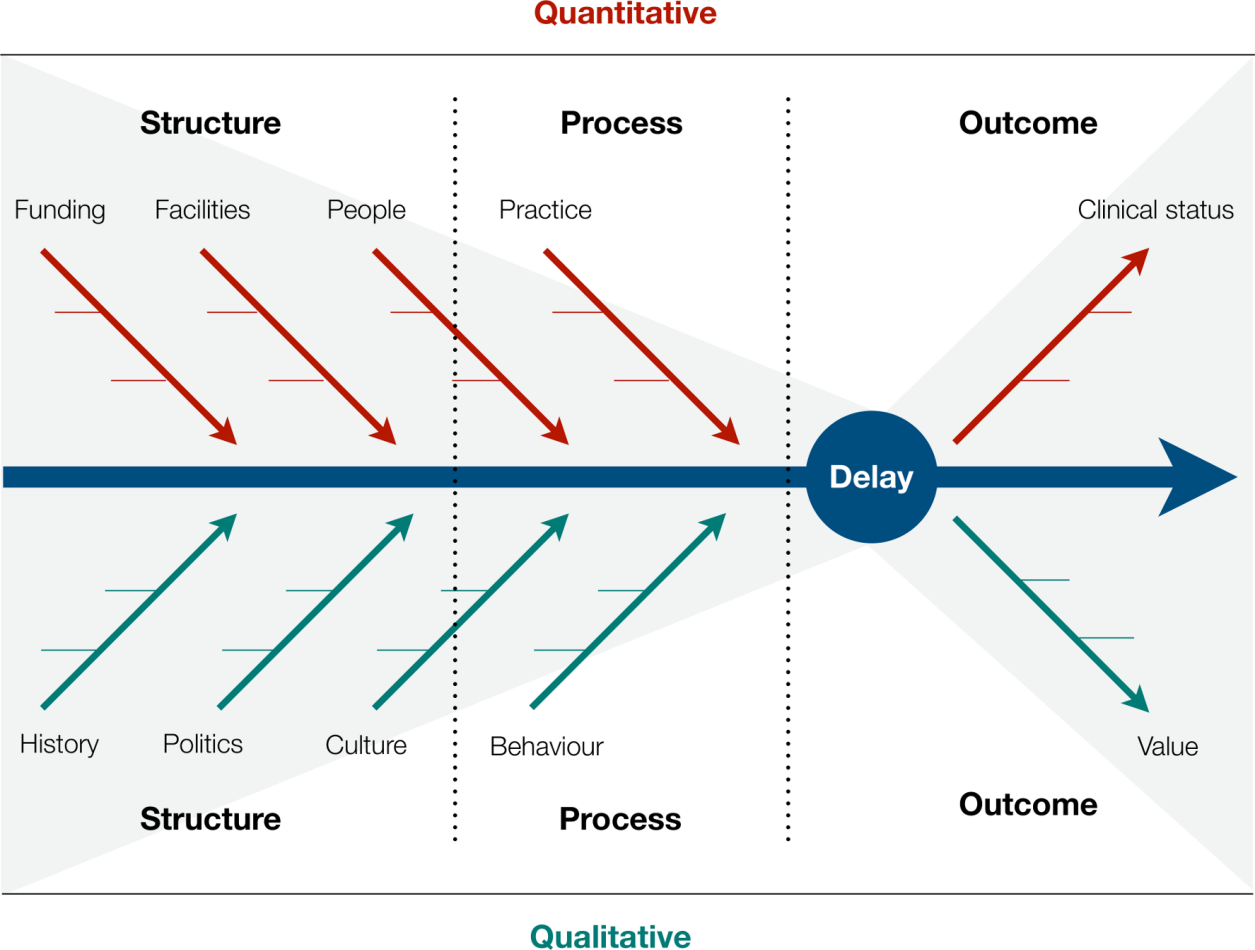
Figure 1Modified Ishikawa diagram showing how multiple factors may contribute to the causes and effects of delay, structured using a Donabedian model of health system function. The horizontal division demonstrates how quantitative and qualitative approaches may explore different factors, while the grey shading indicates that although these factors can be delineated, they are components of a complex web of interdependent elements.

However, understanding the temporal functioning of a health system is challenging, with both quantitative and qualitative approaches required to explore a health system’s problems, create potential solutions and evaluate their effects (figure 1).^7^ Quantitatively, it requires good quality data at multiple time points, coupled with appropriate modelling techniques, to identify gaps and measure improvements in response to interventions. Qualitatively, it requires expertise in methodologies which allow the lived experience of multiple stakeholders to be elicited, understood and integrated into a shared understanding of how decisions are made and their impact on the time taken to receive care. Health systems are complex, with delays in care an emergent phenomenon of reciprocally interacting people, equipment, institutions, processes and cultures (figure 1). Given this, it is unsurprising that designing and evaluating pragmatic interventions to improve speed have proved difficult even in high-resource healthcare environments.^8^

How can this be remedied in the context of resource-poor settings? Time series data may be lacking from existing datasets but can be readily incorporated into prospective surveys or registries, which are gaining ground in global health research.^9^ These data then need to be incorporated into appropriate models, which in turn need to be informed by local context, and accessible to local researchers.^7^ Qualitative understanding may present a greater challenge to those steeped in medical science and is likely to require collaboration with others versed in fields such as ethnography, design, phenomenology or actor-network theory. Combining qualitative and quantitative understanding into practical interventions is a further challenge, and may benefit from engagement with fields such as implementation science or systems engineering.

These different approaches need to then be synthesised to address both context-specific and more generalisable questions. How can the trade-off of speed against quality, acceptability and economic cost be estimated? How can convergent and divergent social, historical and political factors be managed? How can lessons learnt in one setting (either high or low income) be applied to another? The solution to these problems is likely to lie in collaboration. International research partnerships may help achieve this, by providing a platform for academics, spread across a range of countries and contexts, to explore approaches to these problems while developing mutual research capacity.^10^ We represent partnerships of researchers from both high-income and low-income settings who are committed to addressing these challenges in specific diseases in particular countries. Our experience, however, is that these are mutual problems requiring mutual solutions.
